# Supplementary material for: MetaRibo-Seq measures translation in microbiomes
Source: Nat Commun. 2020 Jun 29;11:3268. doi: 10.1038/s41467-020-17081-z (PMC7324362; doi:10.1038/s41467-020-17081-z)
Supplement: Supplementary file 10 — Supplementary Data 7 [file 41467_2020_17081_MOESM10_ESM.zip › File2/Confidence_VeryHigh_Taxonomy/283143_out.krona.html]

Javascript must be enabled to view this page.

members
magnitude
magnitudeUnassigned
count
unassigned
taxon
rank

283143\_out

7

7
2
superkingdom

1
1224
phylum

1
68525
subphylum

1
29547
class

order
213849
1

1
72293
family

1
209
genus

species

SRS048060\_contig\_number\_contig-100\_10345.50374
1905759
1

6
1239
phylum

6
186801
class

6
186802
order

1897049
6
species

SRS014979\_contig\_number\_contig-100\_89.198089SRS065504\_contig\_number\_38058SRS077194\_contig\_number\_12809SRS143991\_contig\_number\_28795SRS144183\_contig\_number\_contig-100\_567.214444SRS148721\_contig\_number\_42635
